# Dolutegravir induces FOLR1 expression during brain organoid development

**DOI:** 10.3389/fnmol.2024.1394058

**Published:** 2024-05-17

**Authors:** Carlo Donato Caiaffa, Gabriel Tukeman, Christian Zevallos Delgado, Yogeshwari S. Ambekar, Taye T. Mekonnen, Manmohan Singh, Victoria Rodriguez, Emily Ricco, Daniel Kraushaar, Salavat R. Aglyamov, Giuliano Scarcelli, Kirill V. Larin, Richard H. Finnell, Robert M. Cabrera

**Affiliations:** ^1^Center for Precision Environmental Health, Department of Molecular and Cellular Biology, Baylor College of Medicine, Houston, TX, United States; ^2^Dell Pediatric Research Institute, University of Texas at Austin, Austin, TX, United States; ^3^Department of Mechanical Engineering, University of Houston, Houston, TX, United States; ^4^Genomic and RNA Profiling Core, Baylor College of Medicine, Houston, TX, United States; ^5^Fischell Department of Bioengineering, University of Maryland, College Park, MD, United States; ^6^Department of Molecular and Cellular Biology, Molecular and Human Genetics and Medicine, Baylor College of Medicine, Houston, TX, United States

**Keywords:** dolutegravir, neural tube defects, brain organoids, neurulation, HIV

## Abstract

During the first month of pregnancy, the brain and spinal cord are formed through a process called neurulation. However, this process can be altered by low serum levels of folic acid, environmental factors, or genetic predispositions. In 2018, a surveillance study in Botswana, a country with a high incidence of human immunodeficiency virus (HIV) and lacking mandatory food folate fortification programs, found that newborns whose mothers were taking dolutegravir (DTG) during the first trimester of pregnancy had an increased risk of neural tube defects (NTDs). As a result, the World Health Organization and the U.S. Food and Drug Administration have issued guidelines emphasizing the potential risks associated with the use of DTG-based antiretroviral therapies during pregnancy. To elucidate the potential mechanisms underlying the DTG-induced NTDs, we sought to assess the potential neurotoxicity of DTG in stem cell-derived brain organoids. The gene expression of brain organoids developed in the presence of DTG was analyzed by RNA sequencing, Optical Coherence Tomography (OCT), Optical Coherence Elastography (OCE), and Brillouin microscopy. The sequencing data shows that DTG induces the expression of the folate receptor (FOLR1) and modifies the expression of genes required for neurogenesis. The Brillouin frequency shift observed at the surface of DTG-exposed brain organoids indicates an increase in superficial tissue stiffness. In contrast, reverberant OCE measurements indicate decreased organoid volumes and internal stiffness.

## Introduction

The most effective approach to treating HIV infection is combination antiretroviral therapy (ART), which suppresses the viral load in HIV-seropositive patients, preventing development of AIDS and minimizing the risk of transmission (Phillips et al., 2019; Kanters et al., 2020; Punekar et al., 2021; Dorward et al., 2023). In 2018, data from the Tsepamo birth outcomes surveillance study in Botswana reported an increased risk for neural tube defects (NTDs) among newborns in HIV-seropositive mothers exposed to a DTG-based ART around the time of conception (Zash et al., 2018). Three years later, two cases of NTDs, also associated with DTG exposure at conception, were reported in Brazil (Kreitchmann et al., 2021). Following these findings, the World Health Organization and the U.S. Food and Drug Administration issued guidelines highlighting the potential risks of administering DTG during pregnancy (Vannappagari and Thorne, 2019; World Health Organization WHO, 2019; Panel on Antiretroviral Therapy and Medical Management of Children Living with HIV, 2024).

DTG-containing therapies are a combination of selected medications, which can include lamivudine and abacavir, both with potential inhibitory activity on the viral nucleoside reverse transcriptase, or DTG combined with rilpivirine, a non-nucleoside reverse transcriptase inhibitor (Cento and Perno, 2020; Panel on Antiretroviral Therapy and Medical Management of Children Living with HIV, 2024). These DTG-containing therapies provide a high barrier to drug resistance, fewer drug–drug interactions, and lower toxicity at a more affordable cost than other ARTs. The WHO has recommended these combinations since 2016 as the preferred first and second-line therapies for all HIV-seropositive patients (Radford et al., 2019; World Health Organization WHO, 2019; Punekar et al., 2021).

The risk period for developing NTDs occurs early during pregnancy, at approximately the fourth-week post-fertilization. The initial outcome of the Tsepamo study reported a total of four infants born with NTDs in a group of 426 women adhering to a DTG-based ART around the time of conception, representing a relative NTD risk of 0.94% (Zash et al., 2018). This early signal for NTDs was reevaluated in 2019 with a follow-up measure of additional births, including five newborns presenting with NTDs in a total of 1,683 deliveries, indicating a decrease in prevalence to 0.30% among the DTG-exposed group (Zash et al., 2019). In a more recent update from March 2022, ten infants presenting NTDs were reported in a group of 9,460 women exposed to DTG-ART at conception, decreasing the NTD risk to 0.11% (Zash et al., 2022). According to the WHO pharmacovigilance database (VigiBase), a total number of 17 NTD cases were reported after exposure to a DTG-ART in a period ranging from 2012 to September 30, 2019. Based on this information and using a case-non-case statistical approach considering the number of NTDs after exposure to a DTG-ART, compared with the number of NTDs associated with all other ARTs reported on VigiBase, Chouchana and colleagues reported that the odds ratio of NTDs related to DTG exposure was 6.4 (95% CI 3.7–10.9). However, when specifically comparing the number of NTDs reported after a DTG-ART using the efavirenz regimen as a reference, the odds ratio was 10.4 (4.9–21.6) (Chouchana et al., 2020).

Besides the observed decline in prevalence for NTDs associated with DTG exposure at conception, it is still important to understand mechanistic interactions between DTG-containing therapies and NTD risk. An explanation for the initial alert might be related to the fact that Botswana is a country with high levels of HIV infection in the absence of a food folate fortification program. Folate-responsive NTDs are a preventable cause of morbidity and mortality globally because folate is the most significant known nutritional modifier of NTD risk and is an essential nutrient required for normal neural tube closure and development. Folate deficiency is consistently associated with increased risks of embryos developing NTDs during the first month of human pregnancy (Finnell et al., 2021; Crider et al., 2022; Finnell and Zhu, 2023; Han et al., 2024).

During the DolPHIN-1 (dolutegravir in pregnant HIV mothers and their neonates) randomized trial in Uganda and South Africa, DTG was detected in samples of breastmilk and placenta and was associated with slower fetal drug metabolism leading to significant exposure levels in the infant plasma (Waitt et al., 2019). We have previously reported a correlation between low serum folate levels and an elevated susceptibility to NTDs in mice exposed *in utero* to DTG (Tukeman et al., 2023). Binding studies have also shown that DTG can act as a non-competitive and partial antagonist of FOLR1. Remarkably, exposure of zebrafish embryos to DTG resulted in developmental delays, which were mitigated by folate supplementation (Cabrera et al., 2019). Multiple mouse studies support a causal relationship between DTG exposure at therapeutic levels and an increased risk for NTDs analogous to those observed in the Tsepamo study (Mohan et al., 2021, 2023; Tukeman et al., 2023). In a morphogenesis model derived from mouse pluripotent stem cells, DTG exposure inhibited growth and axial elongation during neurodevelopment, altering the expression profiles of genes associated with embryonic patterning regulation (Kirkwood-Johnson et al., 2021). DTG exposure at subtherapeutic concentrations in the H9 human embryonic stem cell line (hESCs) and Ca1S line also interfered with the expression of genes regulating early differentiation, decreasing the number of hESCs and pluripotency (Smith et al., 2022).

To investigate the potential mechanisms behind NTDs associated with DTG-containing therapies at conception, we aimed to model the influence of DTG exposure during early embryonic development of the central nervous system using a well-established stem cell-derived brain organoid differentiation protocol. These models are three-dimensional cellular structures employed to analyze human brain development *in vitro* and assess the potential neurotoxicity of drugs and other compounds during early neural development (Lancaster et al., 2013; Lancaster and Knoblich, 2014; Zheng et al., 2021).

The effects of DTG exposure during brain organoid development were quantified in the earliest stages of organoid maturation by high throughput RNA sequencing and by the measurement of biomechanical disturbances using multimodal optical instruments, including Optical Coherence Tomography (OCT) and Brillouin microscopy or Optical Coherence Elastography (OCE) to acquire structural images associated with mechanical mapping.

## Materials and methods

### Human stem-cell-derived brain organoids culture conditions

**Figure 1:**
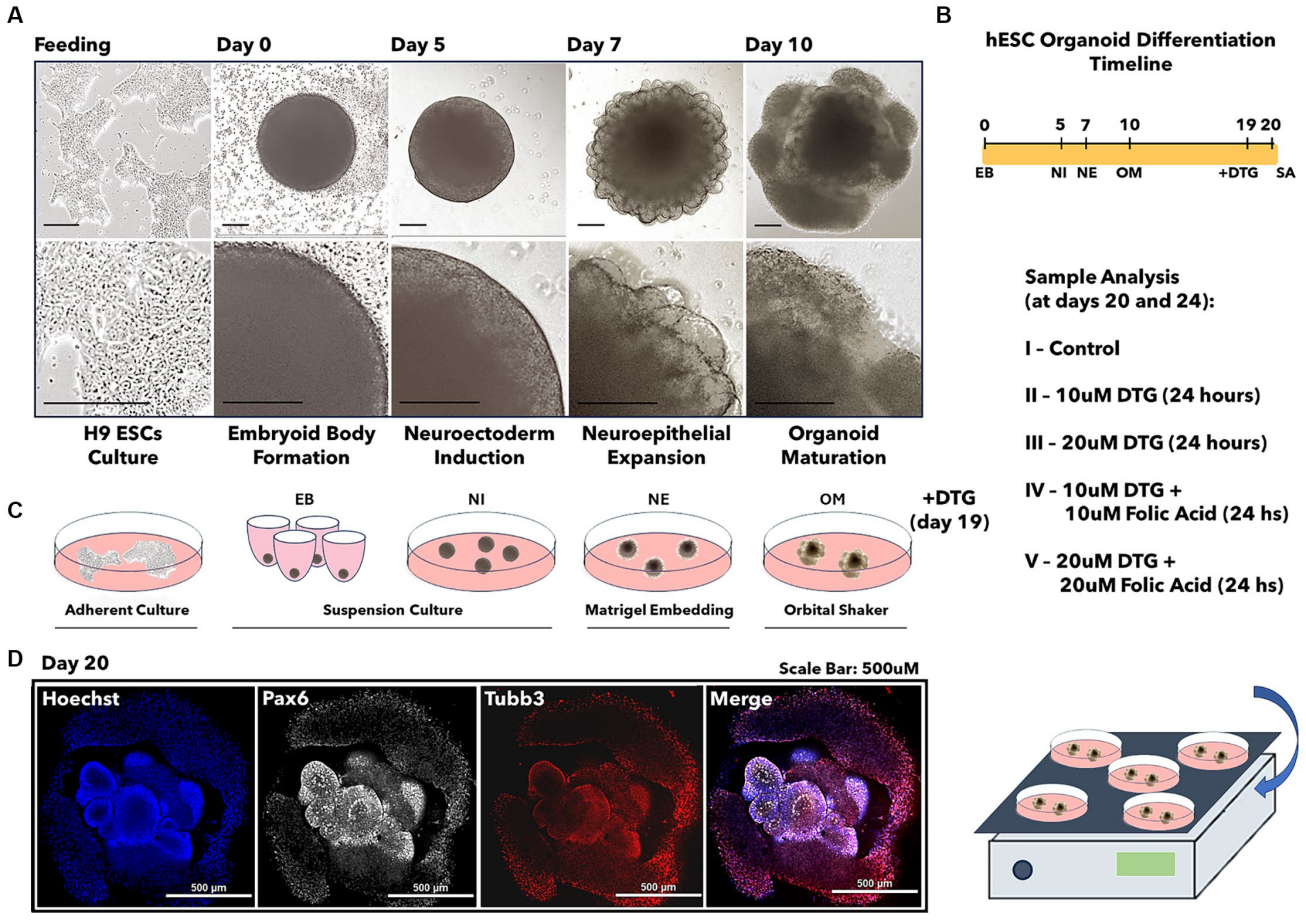
Illustration of the brain organoid development prior to dolutegravir exposure. Light microscopy images representing the differentiation steps from stem cells into brain organoids **(A)**. Brain organoid differentiation timeline **(B)**. Illustration of cell culture passages required for brain organoid development **(C)**. Confocal imaging representing the immunostaining for the differentiation markers Pax6 and Tubb3 **(D)**. The scale bar is 0.5 mm.

Human H9 embryonic stem cells (WA09) were obtained from WiCell. The cell lineage was previously verified for pluripotency, genotype, and mycoplasma-free content and was fed in mTesr Plus media for stem cell culture (Stem Cell Catalog # 100–0276) during at least two passages before differentiation. The Stem Cell Technologies Cerebral Organoid kit (Stem Cell Catalog # 08570) generated brain organoids. In summary, the formation of embryoid bodies (EBs) was induced by the passage of a single cell suspension plated with EB media and 50 μM Y-27632 ROCK inhibitor for 2 days into a 96-well low attachment, U-bottom plate, followed by 3 days of feeding in EB media alone. As previously described, the single-cell suspension was derived from the hESCs colonies cultured in a single well from a six-well plate (Lancaster et al., 2013; Lancaster and Knoblich, 2014). On day 5, the EB media was replaced with Neural Induction media. On day 7, EBs were embedded in 20 μL matrigel droplets using imprinted microwells on parafilm. Matrigel embedded EBs were then incubated for 20 min at 37°C and transferred to a new six well plate containing small groups of organoids in the presence of 3 mL of Neuroepithelial Expansion media per well and kept for 3 days. On day 10, the brain organoids were placed in a shaker inside an incubator and fed with organoid maturation media every 3 days. For DTG exposure experiments, 10 μM DTG, 20 μM DTG, 10 μM DTG, and 10 μM Folic acid or 20 μM DTG and 20 μM Folic acid were added to the maturation media from day 19 until day 20 (Figure 1). The organoid maturation media feeding the organoids in the control group was prepared with an equivalent volume of DPBS containing 1% FBS.

### Immunohistochemistry and DAPI staining

Organoids were fixed in 4% paraformaldehyde (PFA) during 4 h at 4°C. Pax6 (42–6,600, ThermoFisher) and Tubb3 (2H3-Tuj1, DSHB) whole-mount immunostaining was performed on brain organoids in TBS containing 0.1% Tween-20 (TBST) and incubated in a 1:500 dilution of anti-Pax6 and anti-Tuj1 overnight at room temperature. Brain organoids were washed five times in PBST and then incubated with 1:1000 dilution of fluorescent secondary antibodies. For general cell visualization, the brain organoids were incubated overnight in DAPI (4′,6-diamidine-2-phenylidole-dihydrochloride, 2 μg/mL) to label all the nuclei. Brain organoids were cleared in 25% glycerol and imaged using a Nikon CSU-W1 Yokogawa spinning disc confocal microscope. The obtained Z-stacks were projected at maximum intensity and exported as TIFF files.

### High throughput RNA-sequencing

#### RNA-seq library construction

Total RNA samples were normalized to approximately 1 ng each, based on Agilent Bioanalyzer or RiboGreen quantitation. The Takara SMARTer^®^ Stranded Total RNA-Seq Kit v3 – Pico Input Mammalian (p/n 634,485) was used to generate Illumina-compatible libraries for sequencing. The Switching Mechanism at 5′ end of RNA template (SMARTer) cDNA synthesis technology generates directionally identifiable ds-cDNA, incorporated with Illumina sequencing adapters and sample barcodes. Following cDNA synthesis and purification, ribosomal cDNA reduction is achieved using probes (R-Probes v3) targeting mammalian ribosomal cDNA and human mitochondrial ribosomal cDNA and ZapR v3 cleaving these fragments. The final library is ready for quantification and sequencing after amplifying the library using universal primers and a final purification using Takara NucleoMag NGS Clean-up and Size Select beads. Read 1 sequenced reads map to the antisense strand of the original RNA.

The resulting libraries were quantitated by PicoGreen, and fragment size was assessed with the Agilent 2,100 Bioanalyzer. All samples were pooled equimolarly and re-quantitated by qPCR using the Applied Biosystems ViiA7 Quantitative PCR instrument and a KAPA Library Quant Kit (p/n KK4824) and re-assessed on the Bioanalyzer.

#### Cluster generation by exclusion amplification (ExAMP)

Using the concentration from the ViiA7 TM qPCR machine above, 325 pM of equimolarly pooled library is loaded onto one lane of the NovaSeq SP flowcell (Illumina p/n 20,028,400) following the XP Workflow protocol (Illumina kit p/n 20,021,664) and amplified by exclusion amplification onto a nanowell-designed, patterned flowcell using the Illumina NovaSeq 6,000 sequencing instrument. PhiX Control v3 adapter-ligated library (Illumina p/n FC-110-3001) is spiked-in at 10% by weight to ensure balanced diversity and to monitor clustering and sequencing performance. A paired-end 150 bp cycle run was used to sequence the flowcell on a NovaSeq 6,000 Sequencing System. An average of 80 million read pairs per sample was sequenced.

FastQ file generation was executed using Illumina’s cloud-based informatics platform, BaseSpace Sequencing Hub.

### Multimodal Brillouin-Optical Coherence Tomography integrated system

The multimodal Brillouin-OCT system consists of swept source OCT coupled to Brillouin microscopy with a dual virtually imaged phase array (VIPA) spectrometer and a combined scanning arm. The Brillouin utilizes a 660 nm laser source with incident power on the sample at 35 mW. The detected backscattered light from the sample was then transferred to a dual VIPA spectrometer. An electron-multiplying charge-coupled device camera was used to detect the Brillouin frequency shift of the sample. The camera acquisition time was set at 0.2 s. The system calibration was performed before every organoid analysis using standard reagents such as ultrapure water, acetone, and methanol. Every sample was imaged using an achromatic doublet with 0.25 NA to achieve an axial resolution of ~36 μm and lateral resolution of ~3.8 μm. The swept source OCT sub-system had a central wavelength of ~1,310 nm, a scan rate of 50 kHz, a scan range of ~105 nm, and ~ 8 mW incident power on the sample. The lateral and axial resolutions were ~ 17.5 μm and ~ 10 μm in air, respectively. Light from both systems was combined using a dichroic mirror, and galvanometer-mounted mirrors scanned the beam across the sample. For Brillouin imaging, the sample was stepped by a manual vertical stage. A custom software was developed to utilize the OCT structural imaging to guide Brillouin exposition.

### Reverberant optical coherence elastography system

Reverberant Optical Coherence Elastography (Rev-OCE) experiments were performed using organoids exposed to DTG and Folic Acid in different concentrations and two growth stages (20 and 24 days). For each growth stage, five different treatment groups were analyzed and defined as control (regular growth media), DTG 10 μΜ, DTG 20 μΜ, DTG 10 μΜ plus Folate 10 μΜ, and DTG 20 μΜ plus Folate 20 μΜ, and three samples per group were analyzed. The samples were placed on a 35 mm x 10 mm plate filled with 1% agarose (Sigma-Aldrich). The Rev-OCE experiments used a phase-sensitive Optical Coherence Tomography (PhS-OCT) system. On the PhS-OCT, the central wavelength of the light source was 840 nm, the lateral resolution was ~8 um, and the axial resolution in air was ~9 um. The displacement stability was 0.28 nm. The experiments were done with a 25 kHz A-line acquisition rate (temporal resolution ∆𝑡=40 𝑢𝑠). For Rev-OCE, a 3D-printed ring with eight equidistant rods attached to a piezoelectric actuator (BA4510, PiezoDrive) produced a reverberant field in the organoids. The organoids were placed in the middle of the agarose plate, while the eight rods were placed at an equidistant distance surrounding the sample (~2 mm). A 10-pulse quasi-harmonic signal with a frequency of 3 kHz was generated for the Rev-OCE acquisition. The sinusoidal signal was created by the function generator (DG4162, RIGOL Tech, Beijing, China) and amplified by a power amplifier (PDu150, PiezoDrive). 3D M-C mode scans of 500 A-lines were acquired at 101 by 101 points varying from 2 × 2 mm2 to 2.8 × 2.8 mm2 (x-axis and y-axis, respectively) according to the size of the organoids. The phase shift (∆𝜑) on sequential A-lines to estimate the axial particle velocity (𝑣𝑧) was calculated using the equation 𝑣𝑧 = 𝜆0∆𝜑/(4𝜋𝑛∆𝑡) where 𝑛=1.34 (refractive index for brain tissue) [4]. The wave speed was filtered using a 2D spatial bandpass filter to minimize the noise, and the local shear wave speed was determined by 𝑣𝑠 =2𝜋𝐹/𝑘, k was the local wavenumber determined by the 2D autocorrelation profiles from the reverberant analytical solution with a windows size of 0.5×0.5 mm^2^, and the frequency of excitation (𝐹). The volumetric calculations of the organoids were computed using the average (horizontal and vertical) of the diameter (𝑑) for each sample. The calculation was determined by 𝑉_𝑜𝑟𝑔𝑎𝑛𝑜𝑖𝑑_ = 4/3𝜋r^3^ [6], assuming the sphericity of each organoid.

## Results

### Development of human brain organoids to model dolutegravir exposure at conception

To test the hypothesis that newborns present with NTDs after exposure to a DTG-based antiretroviral therapy administered concurrent with neurulation, we differentiated human stem cells (H9 – WiCell) into brain organoids (Lancaster et al., 2013; Lancaster and Knoblich, 2014) during a total period ranging from 20 to 24 days in culture (Figure 1). The protocol for differentiating human embryonic stem cells into brain organoids consists of four successive differentiation steps beginning with the formation of embryoid bodies during 5 days. The second step induces a neuroectodermal fate in the embryoid bodies for 2 days, prior to a consecutive step of neuroepithelial expansion in Matrigel droplets for 3 days. The final differentiation step promotes the maturation of brain organoids after the tenth day in culture. At the beginning of the organoid maturation stage, groups of 20 to 25 organoids were transferred into each well of a six-well plate and then cultured in organoid maturation media for an additional 9 days. On the 19th day of culture, the organoids grown in each well were defined as control groups or four separate groups, which were used to investigate the effects of DTG exposure.

The therapeutic serum concentrations of DTG were previously described to be in the range of 3 to 10 μΜ (Eron et al., 2013; Castagna et al., 2014). In a previous report, we showed that binding of folate to FOLR1 was reduced 54% by 12 μM DTG (Cabrera et al., 2019). Based on these findings, the organoids in the experimental group were exposed for 24 h in organoid maturation media containing dolutegravir (DTG 10 μM or DTG 20 μM diluted in DPBS with 1% FBS) or a combination of dolutegravir and folic acid (DTG 10 μM plus FA 10 μM or DTG 20 μM plus FA 20 μM diluted in DPBS with 1% FBS) (Figures 1A–C). An equivalent volume of DPBS with 1% FBS was added to the organoid maturation media in the control group. On the 20th day in culture, representative samples from each group were immunostained to detect the presence of the markers Pax6 and Tuj1, confirming differentiation of the pluripotent cells into a neural progenitor lineage (Figure 1D), followed by high-throughput RNA sequencing (RNA-seq) or physical analysis of structural and biomechanical properties.

### Dolutegravir exposure modifies the gene expression patterns during brain organoid development

An unbiased and comprehensive analysis was performed following DTG exposure, or a combination of DTG and FA, on gene expression patterns during brain organoid development. The expression of all the transcribed messenger RNAs present was analyzed from a pool of 20 organoids per experimental group. To reduce the high dimensionality of the bulk RNA-seq result and identify the statistically significant variations in the data, we applied principal component analysis (PCA) on the brain organoid RNA-seq datasets. The broad impact of DTG exposure on brain organoids is represented by the principal component 1 (PC1). In contrast, the role of FA in response to DTG exposure is represented by the principal component 2 (PC2).

**Figure 2:**
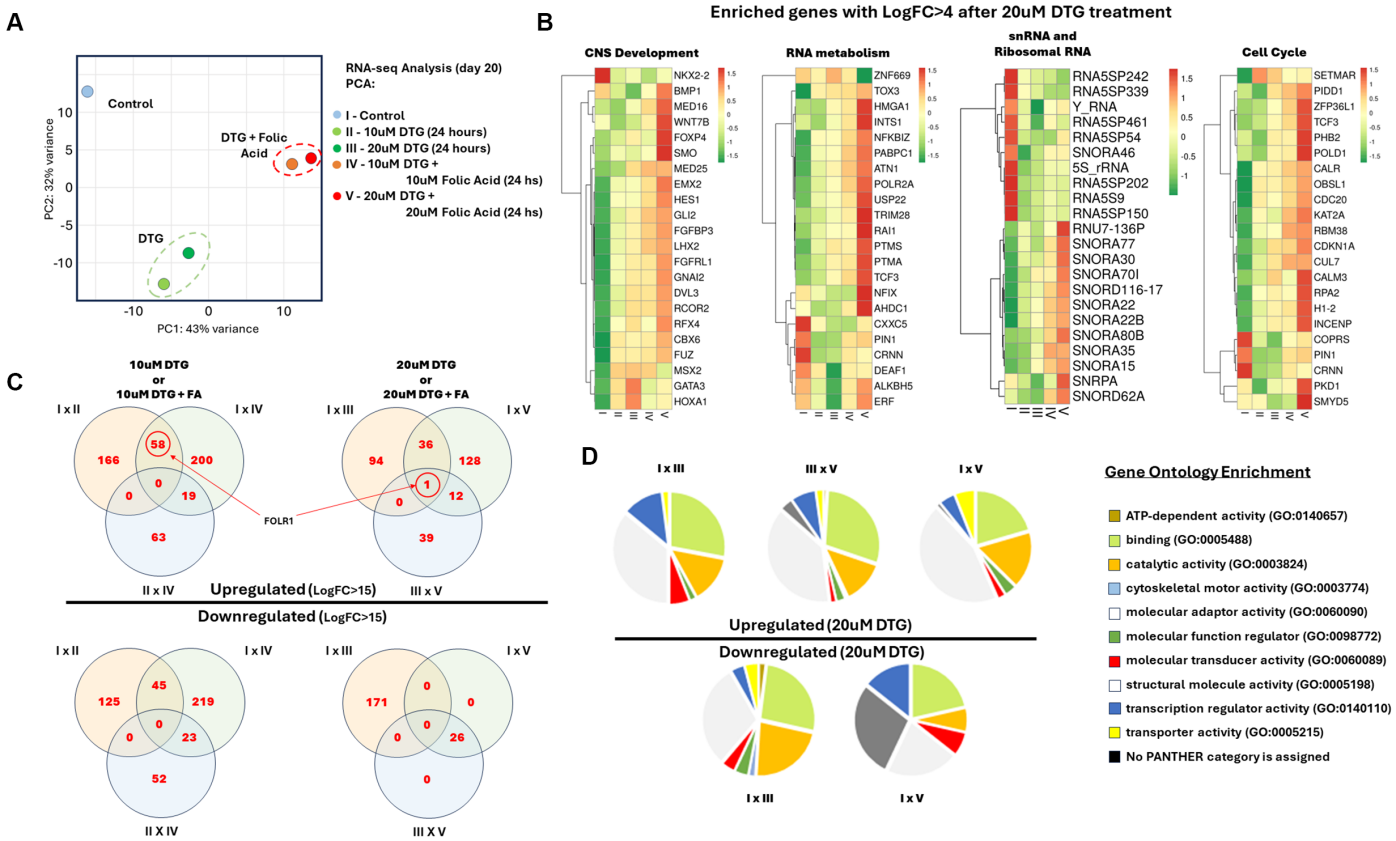
Identification of FOLR1 as a highly expressed transcript in brain organoids exposed to DTG. Illustration of the sample preparation steps prior to RNA sequencing and analysis **(A)**. RNA-seq Principal Component Analysis **(B)**. Heatmaps of enriched gene ontologies presenting LogFC>4 after exposure to 20 μΜ DTG **(C)**. Venn diagrams comparing the differential gene expression in between the experimental groups **(D)**. Pie charts representing the gene ontologies upregulated and downregulated after exposure to 20 μΜ DTG **(E)**.

Regarding variations related to the dominant data gathered by both PCA components, organoids exposed to DTG diverge from the control, presenting distinctive higher PC1 values and a lower PC2. Beyond the particularly lower PC1 and higher PC2 values found in the control, the negative PC2 values found in brain organoids exposed to DTG indicate a distinct change in the gene expression patterns after DTG exposure. When brain organoids were exposed to DTG in the presence of FA, the increased values detected in both the PC1 and PC2 axes support a protective role of FA, attenuating the gene expression shift observed in brain organoids exposed to DTG (Figure 2A).

### DTG modifies the gene expression pattern required for normal neural development

To visualize the impact of DTG exposure on brain organoids, we performed differential gene expression analysis on the RNA-seq landscape measured in all groups exposed to DTG or DTG and FA. The biological mechanisms underlying alterations in gene expression were further analyzed using the Panther and ToppFun gene ontology enrichment tools (Chen et al., 2009; Mi et al., 2021). Figure 2C reports the transcriptomic changes identified in genes regulating the cell cycle, RNA metabolism, and central nervous system development, representing a distinct effect observed in all the DTG exposure groups. Another contrasting effect observed in the DTG-exposed groups is transcriptomic changes in an enriched group of small nucleolar RNAs (snRNA) and ribosomal RNAs (Figures 2B,D).

### FOLR1 is highly expressed in human brain organoids exposed to dolutegravir

**Figure 3:**
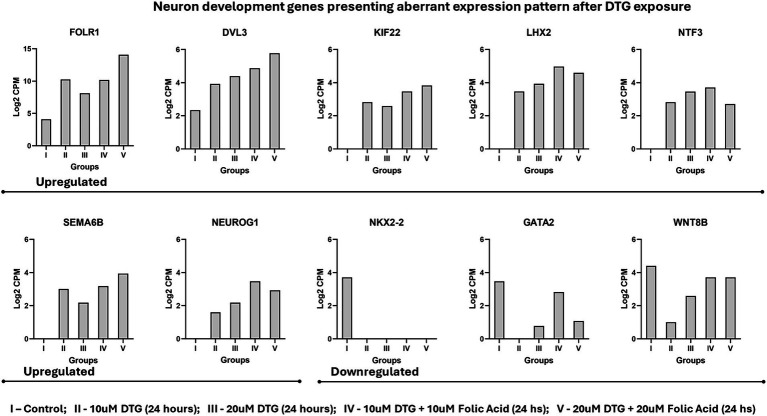
Neuron development genes presenting aberrant expression pattern after DTG exposure. The experimental groups were represented as I – control; II – 10μΜ DTG (24 h); III – 20 μΜ DTG (24 h); IV – 10 μΜ DTG + 10 μΜ Folic Acid (24 hs); V – 20 μΜ DTG + 20 μΜ Folic Acid (24 h).

The Venn diagrams in Figure 2C compare differentially expressed genes presenting a fold change higher than 15. FOLR1 is identified as a highly expressed transcript when comparing the organoids grown in regular maturation media versus organoids exposed to 10 μΜ DTG, 20 μΜ DTG, 10 μΜ DTG plus 10 μΜ FA, or 20 μΜ DTG plus 20 μΜ FA. To eliminate the influence of differing sequencing depths between samples, we used counts per million (CPM) to compare gene expression levels across the samples. As the expression of FOLR1 measured in the control spanned several orders of magnitude in the DTG and the DTG plus FA exposed groups, log2CPM was calculated to represent the wide data range found and to emphasize the detected fold changes in the graph shown in Figure 3.

Considering the biological processes of head, brain, neuron, and neural tube development (GO:0048666), the genes DVL3, GATA2, KIF22, LHX2, NEUROG1, NKX2-2, NTF3, SEMA6B, and WNT8B also represent a specific set of alterations in the gene transcriptional patterns measured on the messenger RNA landscape of DTG exposed brain organoids (Figure 3).

### Dolutegravir induces increased stiffness levels at the organoid surface

**Figure 4:**
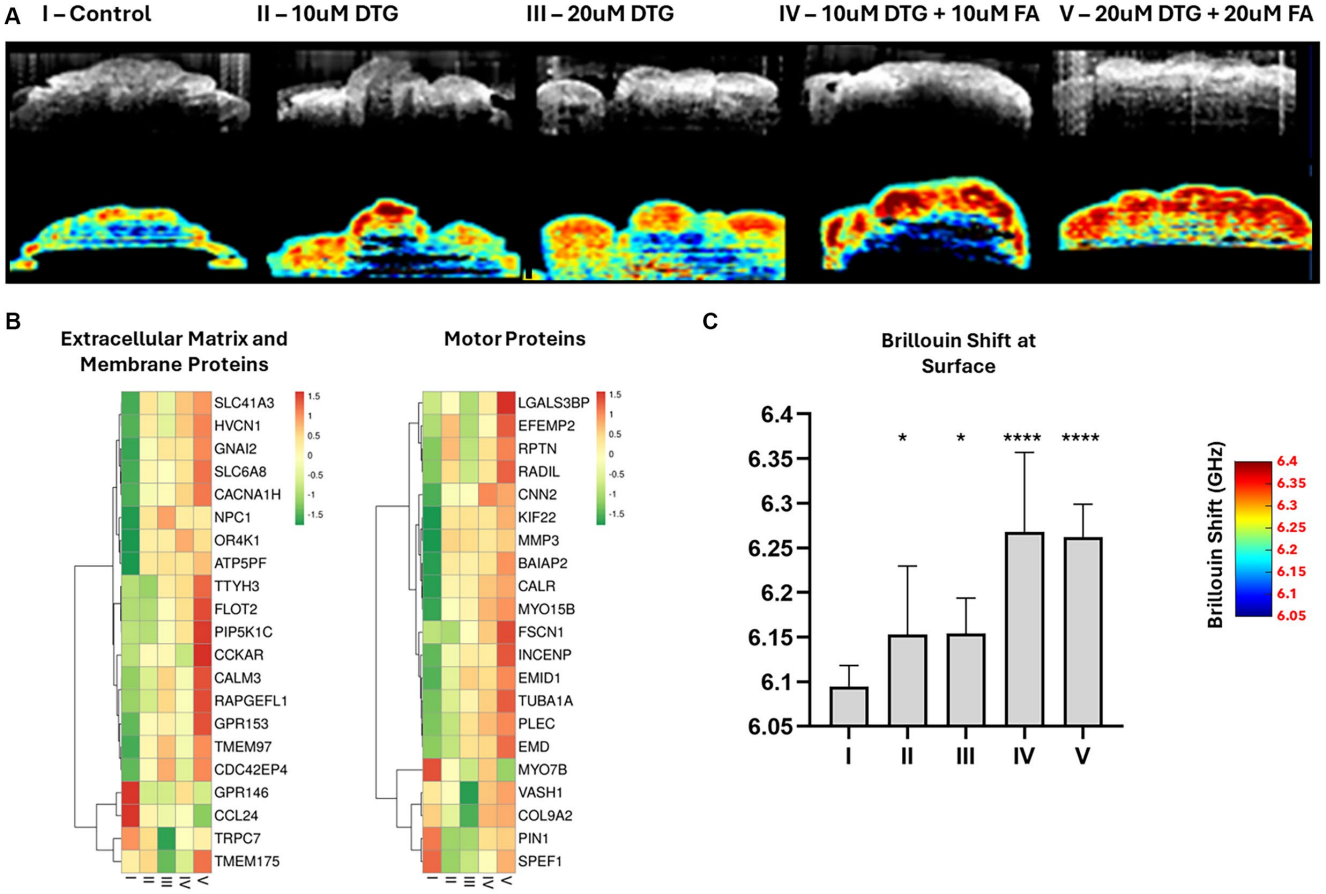
Dolutegravir increases superficial tissue stiffness in brain organoids. Illustration of the multimodal optical system coupling Optical Coherence Tomography with Brillouin microscopy **(A)**. 2D-OCT optical sections and respective Brillouin frequency shift representing superficial tissue stiffness in brain organoids **(B)**. Heatmaps representing an enriched group of extracellular matrix, membrane, and motor proteins disturbed with Log Fold Change >4 after exposure to 20 μΜ DTG **(C)**. Surface-wise average Brillouin frequency shift of brain organoids **(D)**. * and **** indicate *p* < 0.05 and *p* < 0.0001, respectively by One-Way ANOVA and Bonferroni’s comparisons test.

The ability of stem cells to aggregate, forming embryoid bodies (EBs), is a crucial process guided by cell–cell adhesion during organoid development. Once formed, EBs allow the development of methods to differentiate three-dimensional cellular structures featuring cell–cell interactions, signaling pathways, and extracellular matrix contents in a comprehensible replica of human biology. To investigate whether DTG exposure would cause biomechanical alterations to the organoid structure, we used a multimodal imaging technique built on the association of Optical Coherence Tomography (OCT) and Brillouin light scattering to measure superficial stiffness on the brain organoids exposed to DTG. The real-time imaging capability of OCT was used to define a transverse section throughout the brain organoids before a prolonged exposition time and respective detection of the Brillouin light scattering (Figure 4).

To determine if there was a significant difference in the biomechanical properties of organoids exposed to DTG, the Brillouin frequency shift detected at the organoid surface was statistically tested using one-way ANOVA and Bonferroni’s multiple comparisons test. Organoids exposed to DTG presented higher average surface stiffness than the control (Figures 2A,C). The Brillouin frequency shift measured at the surface of brain organoids exposed to both DTG and FA significantly differed from the control or DTG groups, indicating that FA addition escalated the stiffness levels on the surface of these organoids. The increased stiffness levels at the surface of the brain organoids follow the effect of DTG overexpressing FOLR1 and represent the disturbance observed in the expression patterns of an enriched group of motor proteins, membrane components, and extracellular proteins (Figures 3, 4).

### Organoid volume and internal stiffness levels are decreased after exposure to dolutegravir

**Figure 5:**
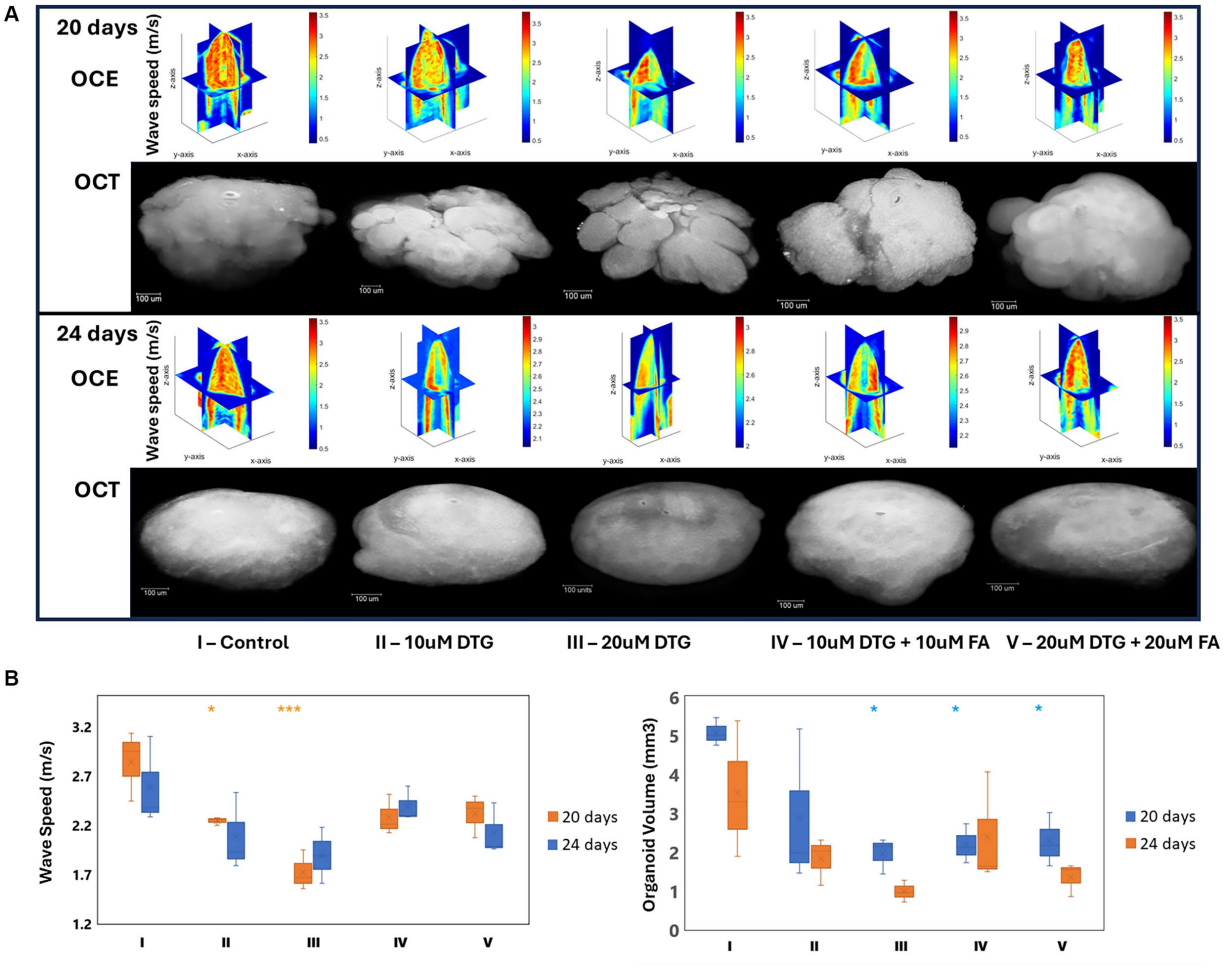
Dolutegravir exposure decreases the organoid core’s volume and stiffness levels. Illustration of the multimodal optical instrument associating Optical Coherence Tomography and reverberant optical coherence elastography **(A)**. 3D elastic wave speed maps and 2D-OCT optical sections representing general tissue stiffness in brain organoids **(B)**. Region-wise average elastic wave speeds and brain organoid volumes **(C)**. *and *** indicate p < 0.05 and *p* < 0.001, respectively by One-Way ANOVA and Bonferroni’s comparisons test.

The distinctive elastic wave speeds and tissue-structural features in representative brain organoids exposed to DTG for 24 h were recorded at 20 or 24 days *in vitro* (Figure 5). A normalized focal three-dimensional intensity map was averaged over parallel measurements of organoids exposed to DTG (Figure 5A). As can be observed from the 3D maps, the elastic wave propagates further laterally in the organoids treated with DTG, while the control group exhibits a faster signal attenuation. The wave speed maps indicate that the inner core of the organoids is less stiff than the surface. While there is plausible dimensional variation in organoid stiffness distribution, the significant differences observed in the graph might result from the increased stiffness in the surface exposed to maturation media containing DTG or a combination of DTG and FA (Figure 5B).

The volumetric calculations of the brain organoids were then quantified using the average diameter obtained for each sample. The estimated volume was determined considering the sphericity of each organoid by a calculation assuming 𝑉_𝑜𝑟𝑔𝑎𝑛𝑜𝑖𝑑_ = 4/3𝜋r^3^. As can be observed in Figure 5B, brain organoids exposed to DTG on the 19th day in culture, for 24 h, presented smaller volumes on day 24 than on day 20, indicating that the effect of DTG delaying organoid growth is extended over time and that the addition of FA does not rescue organoid growth.

## Discussion

The World Health Organization recommends dolutegravir inclusion in both first- and second-line antiretroviral therapies for all individuals living with HIV (World Health Organization WHO, 2019). The mechanism of HIV-1 infection hinges on the enzymatic integration of viral DNA into the host genome, a process mediated by a retroviral integrase. DTG, classified as an integrase strand transfer inhibitor (INSTI), selectively binds to the magnesium ions located at the integrase catalytic domain, inhibiting the integration of viral DNA into the host DNA, a critical step in the viral replication cycle (Hare et al., 2010; Cook et al., 2020).

A growing number of articles published using *in vitro* studies (Cabrera et al., 2019; Zamek-Gliszczynski et al., 2019; Kirkwood-Johnson et al., 2021), animal models (Stanislaus et al., 2020; Mohan et al., 2021, 2023; Tukeman et al., 2023) and in humans (Zash et al., 2018; Money et al., 2019; Vannappagari et al., 2019; Zash et al., 2019; Pereira et al., 2021), are investigating the link between birth defects following exposure to a DTG-based antiretroviral therapy. While debates may continue, environmental factors, genetic predispositions, exposure to certain drugs, and maternal influences, such as serum folate levels, are recognized risk factors for congenital anomalies, including neural tube defects.

Our study aimed to quantify the impact of DTG exposure on the gene expression patterns and tissue biomechanics in human stem-cell-derived brain organoids. We have previously reported that low serum folate levels in mice exposed to DTG cause increased incidences of NTDs (Tukeman et al., 2023). Additionally, we discovered a partial antagonism between DTG and FOLR1, which resulted in developmental toxicity caused by DTG exposure during zebrafish embryonic development that could be rescued by adding folic acid (Cabrera et al., 2019). Our work demonstrates a significant induction of FOLR1 expression in brain organoids after adding DTG to the maturation media for 24 h. FOLR1 upregulation was accompanied by disruptions in the expression patterns of genes associated with normal neuron development, cell cycle, extracellular matrix, membrane, and motor proteins. Furthermore, DTG exposure increased stiffness at the organoid surface and reduced volume during maturation in cell culture.

We utilized Principal Component Analysis (PCA) to reduce the dimensionality and identify significant variations in the RNA-seq datasets. While PC1 represents the broad impact of DTG exposure, PC2 reveals the role of FA in response to DTG during brain organoid maturation. DTG exposure is displayed by a divergence in the gene expression patterns found in the control, increasing the observed PC1 values and lowering PC2 values, indicating a distinct effect of DTG exposure in the gene expression patterns of the brain organoids. Remarkably, the negative PC2 values found in DTG-exposed organoids further emphasize the disturbance in the gene expression. This shift in the gene expression patterns induced by DTG is attenuated when organoids were exposed to DTG in the presence of FA, suggesting a protective role of FA in this group, as indicated by an increase in values for both PC1 and PC2.

To better understand how DTG or a combination of DTG and FA influence the expression of genes in brain organoids, we used the Comparative Marker Selection tool available on the GenePattern server (Reich et al., 2006). The Venn diagrams illustrated in Figure 2D highlight genes with a fold change higher than 15, and it is possible to observe the presence of FOLR1 emerging as a highly expressed gene in organoids exposed to different concentrations of DTG or DTG plus FA.

When measuring the gene expression in brain organoids exposed to DTG or the combination of DTG and FA, the expression of FOLR1 quantified in the control spanned multiple orders of magnitude in the DTG exposure groups. To represent this wide expression range of FOLR1 across multiple orders of magnitude, we employed the calculation of log2CPM to effectively capture and highlight the extensive data range observed in Figure 3, thereby providing a more explicit representation of the FOLR1 expression within the experimental conditions.

Folic acid is an essential nutrient required for DNA synthesis, methylation processes, and gene expression during neural tube closure and central nervous system development. The gene FOLR1 encodes the folate receptor alpha, a transmembrane protein presenting nM affinity for binding folic acid (vitamin B9) and reduced folic acid derivatives, mediates cellular folate uptake. Knockdown of *Folr1* in murine mutant models results in embryo lethality and abnormal neural tube development, consequently leading to folate modifiable NTDs (Piedrahita et al., 1999). Disorders affecting folate metabolism and transport can also result in folate deficiencies (Goyette et al., 1994; Leclerc et al., 1996, 1998; Rosenblatt and Fenton, 2001; Hilton et al., 2003; Qiu et al., 2006). For example, cerebral folate deficiency is characterized by a deficiency of 5-methyltetrahydrofolate (5-MTHF) within the cerebrospinal fluid, while folate levels in red blood cells and plasma are within the low normal range. This condition can lead to severe neurological symptoms, including developmental delay, cognitive impairment, movement disorders, and seizures. *De novo* loss of function variants of the gene CIC can contribute to cerebral folate deficiency by downregulating FOLR1 expression (Cao et al., 2021). Autoantibodies can also disrupt folate transport mediated by FOLR1, increasing the risk of NTDs during pregnancy by blocking the folate binding sites on the folate receptor alpha (Cabrera et al., 2008). A recent study reported that FOLR1 upregulation reduces the vesicular stomatitis virus replication cycle, leading to a FA deficiency in both HeLa cells and mice. This antiviral activity was related to FOLR1 overexpression associated with FA deficiency (Wu et al., 2023).

Besides the molecular mechanisms regulating the expression of FOLR1 and the cascade of genes interacting downstream to FOLR1 signaling that are unknown, disturbances in the FOLR1 gene expression patterns influence the availability of folate for DNA methylation, which subsequently affects the epigenetic regulation of genes required for neurogenesis, neural tube, and central nervous system development. As revealed in the messenger RNA landscape of brain organoids exposed to DTG, the genes DVL3, GATA2, KIF22, LHX2, NEUROG1, NKX2-2, NTF3, SEMA6B, and WNT8B shown in Figure 3 also exhibit distinct modifications in their transcriptional profiles. All the genes represented in Figure 3 are required for neuron development. DVL3, LHX2, and NEUROG1 are also crucial for neural crest differentiation, and NKX2-2 and WNT8B are essential components of Sonic Hedgehog signaling and Wnt-Lrp6 pathways, respectively. The aberrant expression levels observed in these genes require additional investigations into potential aberrant histone and DNA methylation patterns caused by deficient folate transport mediated by FOLR1 and respective disruptions in the signaling pathways downstream on FOLR1.

Organoids exposed to DTG also exhibited alterations in the expression levels of small nucleolar RNAs (snRNA) and ribosomal RNAs, as illustrated in Figures 2B,D. These superfamilies contain several gene paralogs that need to be better annotated, and there is a current lack of studies describing their function after drug exposure or during neural ontogeny.

The formation of embryoid bodies is pivotal during organoid development, preserving stem cell pluripotency within three-dimensional aggregates. A structural framework is provided initially by the extracellular matrix, allowing the formation of cell–cell adhesive interactions. This dynamic interplay between the cell adhesions and extracellular matrix contributes not only to the formation of cohesive EBs but also plays a crucial role in cell signaling (Bratt-Leal et al., 2009; Zeevaert et al., 2020). As the embryoid bodies are differentiated towards neural progenitors in a controlled environment, it is possible to dissociate specific effects of a drug in a humanized model of brain development (de Poel et al., 2023; Zarate-Lopez et al., 2023).

The use of multimodal instruments coupling OCT and Brillouin light scattering facilitated the assessment of superficial stiffness in DTG-exposed brain organoids. Transversal sections of the brain organoids were delineated using the OCT rapid-imaging capabilities prior to prolonged exposure times and respective detection of Brillouin light scattering. One-way ANOVA and Bonferroni’s multiple comparisons test were employed to provide insights into the significance of biomechanical changes observed in organoids exposed to DTG. Organoids cultivated in maturation media containing DTG exhibited higher surface stiffness than the control group. The impact of adding a combination of FA and DTG to the maturation media was also explored. Notably, the Brillouin frequency shift measured at the surface of organoids grown in maturation media containing FA and DTG differed significantly from the control and DTG groups. This discrepancy indicates a substantial escalation in stiffness levels in the presence of both FA and DTG. The observed increase in surface stiffness follows the effect of DTG on FOLR1 expression and a respective disturbance in the expression patterns of a specific group of motor proteins, membrane components, and extracellular proteins (Figure 4).

As these findings suggested a link between DTG exposure, altered biomechanics, and changes in gene expression patterns, we investigated these observations further using another multimodal optical instrument. This device associates tissue-structure detection using phase-sensitive Optical Coherence Tomography coupled with reverberant optical coherence elastography to record the elastic wave speeds from organoids in culture.

The recorded elastic wave speeds associated with tissue-structural organization detected in DTG-exposed organoids reveal that the wave speeds propagate laterally instead of centrally, indicating the alteration of biomechanical components compared to the control group. The wave speed maps shown in Figure 5C further highlight these differences in stiffness between the inner core and surface of the organoids. These three-dimensional wave speed maps suggest increased stiffness at the organoid surface exposed to maturation media containing DTG, which could be influenced by adding DTG alone or combined with FA.

The volumetric calculations shown in Figure 5A assumed complete organoid sphericity based on the measured average diameter. Brain organoids exposed to DTG for 24 h, on the 19th day in culture, exhibit smaller volumes at day 24 compared to day 20, indicating that the impact of DTG delaying organoid growth intensifies over time during maturation. Notably, adding FA does not rescue organoid growth, suggesting a limitation of FA mitigating the observed growth delay induced by DTG. These findings also provide valuable insights into the dynamics of biomechanical and volumetric alterations induced by DTG in brain organoids.

While the preliminary data from the Tsepamo study estimated a relative NTD risk of 0.94% after four newborns presented with NTDs in a group of 426 HIV-seropositive women (Zash et al., 2018), further surveillance studies weakened this initial NTD alert (Zash et al., 2019, 2022), but there is increasing evidence that DTG antagonizes FOLR1 (Cabrera et al., 2019; Tukeman et al., 2023), which would mechanistically explain the NTD cases associated with DTG-based therapies around the time of conception.

## Data availability statement

The RNA sequencing data presented in this study have been deposited at NCBI GEO accession GSE263394. Please direct further inquiries to the corresponding author. FastQ file generation was executed using Illumina’s cloud-based informatics platform, BaseSpace Sequencing Hub. Raw data have been deposited at NCBI GEO accession GSE263394. https://www.ncbi.nlm.nih.gov/geo/query/acc.cgi?acc=GSE263394.

## Ethics statement

Ethical approval was not required for the studies on humans in accordance with the local legislation and institutional requirements because only commercially available established cell lines were used.

## Author contributions

CC: Conceptualization, Data curation, Formal analysis, Investigation, Methodology, Resources, Validation, Visualization, Writing – original draft, Writing – review & editing. GT: Formal analysis, Investigation, Methodology, Resources, Visualization, Writing – review & editing. CD: Formal analysis, Methodology, Resources, Validation, Visualization, Writing – review & editing. YA: Formal analysis, Investigation, Methodology, Resources, Validation, Visualization, Writing – review & editing. TM: Methodology, Resources, Writing – review & editing. MS: Methodology, Resources, Software, Writing – review & editing. VR: Methodology, Resources, Writing – review & editing. ER: Methodology, Resources, Writing – review & editing. DK: Data curation, Methodology, Resources, Visualization, Writing – review & editing. SA: Methodology, Resources, Software, Writing – review & editing. GS: Funding acquisition, Methodology, Project administration, Resources, Writing – review & editing. KL: Funding acquisition, Methodology, Project administration, Resources, Supervision, Visualization, Writing – review & editing. RF: Formal analysis, Funding acquisition, Project administration, Resources, Supervision, Visualization, Writing – review & editing. RC: Formal analysis, Funding acquisition, Investigation, Methodology, Project administration, Resources, Supervision, Validation, Visualization, Writing – review & editing.
